# Performance of Genotype Imputation for Low Frequency and Rare Variants from the 1000 Genomes

**DOI:** 10.1371/journal.pone.0116487

**Published:** 2015-01-26

**Authors:** Hou-Feng Zheng, Jing-Jing Rong, Ming Liu, Fang Han, Xing-Wei Zhang, J. Brent Richards, Li Wang

**Affiliations:** 1 Institute of Aging Research, School of Medicine, Hangzhou Normal University, and the Affiliated Hospital of Hangzhou Normal University, Hangzhou, Zhejiang, China; 2 Department of Medicine, Human Genetics, Epidemiology and Biostatistics, Lady Davis Institute for Medical Research, Jewish General Hospital, McGill University, Montreal, Quebec, Canada; 3 Department of Pulmonary, Critical Care Medicine, Peking University People’s Hospital, Beijing, China; 4 Twin Research and Genetic Epidemiology, King’s College London, London, United Kingdom; University of North Carolina, UNITED STATES

## Abstract

Genotype imputation is now routinely applied in genome-wide association studies (GWAS) and meta-analyses. However, most of the imputations have been run using HapMap samples as reference, imputation of low frequency and rare variants (minor allele frequency (MAF) < 5%) are not systemically assessed. With the emergence of next-generation sequencing, large reference panels (such as the 1000 Genomes panel) are available to facilitate imputation of these variants. Therefore, in order to estimate the performance of low frequency and rare variants imputation, we imputed 153 individuals, each of whom had 3 different genotype array data including 317k, 610k and 1 million SNPs, to three different reference panels: the 1000 Genomes pilot March 2010 release (1KGpilot), the 1000 Genomes interim August 2010 release (1KGinterim), and the 1000 Genomes phase1 November 2010 and May 2011 release (1KGphase1) by using IMPUTE version 2. The differences between these three releases of the 1000 Genomes data are the sample size, ancestry diversity, number of variants and their frequency spectrum. We found that both reference panel and GWAS chip density affect the imputation of low frequency and rare variants. 1KGphase1 outperformed the other 2 panels, at higher concordance rate, higher proportion of well-imputed variants (info>0.4) and higher mean info score in each MAF bin. Similarly, 1M chip array outperformed 610K and 317K. However for very rare variants (MAF≤0.3%), only 0–1% of the variants were well imputed. We conclude that the imputation of low frequency and rare variants improves with larger reference panels and higher density of genome-wide genotyping arrays. Yet, despite a large reference panel size and dense genotyping density, very rare variants remain difficult to impute.

## Introduction

Genotype imputation [1] is now an important step in the analysis of genome-wide association (GWA) data. This method allows inferring the genotype of a genetic marker, for example a single nucleotide polymorphism (SNP), which is not directly genotyped, thus providing the evidence for association of this mark. Genotype imputation is particularly useful in meta-analysis of GWA studies, where the results across studies were generated by different genotyping platforms. Since common variants, which mainly identified by GWA studies, explain little of the variances of most common diseases [2, 3], and common variants association might be the synthetic associations arising from rare variants on the same haplotype background [4], the next phase in the genetic mapping of common disease will involve sequencing experiments to identify rare variants associated with disease risk. However, the statistical power to associate rare variants with common disease is poor [5, 6], therefore, imputation of rare variants from genome-wide genotypic arrays offers a cost-efficient strategy to achieve necessary sample sizes, provided that additional samples have been genome-wide genotyped.

There are several programs such as BEAGLE [7], MaCH [8] and IMPUTE2 [9], permitting imputation of untyped variants. All these three imputation methods are developed to infer SNP genotypes by linkage disequilibrium (LD) with typed SNPs based on a reference panel. With the emergence of next-generation sequencing technology and the 1000 Genomes Project, several versions of the haplotype data were released as imputation reference panels: the 1000 Genomes pilot data released on March 2010 (1KGpilot) [10], the 1000 Genomes interim data released on August 2010 (1KGinterim), and the 1000 Genomes phase1 data released on November 2010 and May 2011 [11].

Prior to the 1000 Genomes Project, most GWAS meta-analyses have been run using HapMap haplotypes as reference for imputation, we previously assessed the HapMap-based imputation and found that variants with lower MAF are difficult to impute [12], meaning that low frequency and rare variants were not being comprehensively investigated in previous GWAS meta-analyses. Therefore, a primary goal of this study has been to assess the imputation performance of rare variants from the 1000 Genomes, so that additional GWAS samples can be included in the rare variants association analysis after imputation, thus the statistical power could be improved without substantially increasing costs. We therefore imputed 153 participants, each of whom had genotypes on 3 different genotyping arrays including 317k, 610k and 1 million SNPs, to 3 different releases of the 1000 Genomes reference panels. We assessed the performance of imputation for rare variants across these 9 scenarios.

## Materials and Methods

### GWAS samples and genotyping

This study is nested within the TwinsUK study, a prospective study comprising a total of 12,000 identical and non-identical twins from right across the UK with ages between sixteen and ninety-eight. The study has been approved by the institutional review board (Medical Ethics Committee) of the King’s College London, UK. Over 5654 samples have been genotyped with different Illumina (San Diego, CA, USA) microarray beadchips (HumanHap300 (317k), Human- Hap610Q (610k), 1MDuo and 1.2MDuo 1M (1M)) [13, 14, 15], of which, 2040 are from the 317k, 3461 are from the 610k and 153 are from the 1M.

A subset of individuals from the TwinsUK study was chosen as the study sample for this project. We took the 153 subjects genotyped by 1M platform as the study samples (Supporting information), then extracted 317k and 610k SNPs for these 153 samples to make three GWAS datasets of the same 153 samples (317k, 610k and 1M), by doing this, we make sure that the genotype of the same SNP is always the same, the only difference between the 3 GWAS datasets is the density of the SNP.

### Reference panels used for imputation

Three reference panels are the CEU panel from March 2010 released pilot data of the 1000 Genomes Project (1KGpilot, b36), the EUR panel from August 2010 released interim data of the 1000 Genomes Project (1KGinterim, b37), and all panels from November 2010 and May 2011 released phase1 integrate data of the 1000 Genomes Project (1KGphase1, b37) as reference panels. The CEU panel of 1KGpilot has 112 haplotypes (56 samples) and ~8.5 million SNPs, the EUR panel of 1KGinterim has 566 haplotypes (288 samples) and about 11.5 million SNPs, and the all panels of 1KGphase1 has 2188 haplotypes (1094 samples) and about 37.4 million SNPs. The haplotype reference panels were downloaded from website (http://mathgen.stats.ox.ac.uk/impute/impute_v2.html#reference).

### Genotype Imputation

We used IMPUTE version 2 [9] in this study. The 153 samples from the 3 GWAS datasets (317k, 610k and 1M) were first phased without reference panel, respectively [16], then the resulting haplotypes were used to perform fast imputation from the 3 reference panels. In order to decrease the real computing time, we split each chromosome into ~5M chunks for analysis, the chunks then could be imputed in parallel on multiple computer processors, both phasing and imputation were done by chunks. In total, 558 chunks were obtained in the genome. For 153 samples, it took ~9.5 min to phase a chunk with 30 iterations in a machine with 64GB RAM, and it took ~5 min to impute a chunk from the best guess phased haplotype.

Basically, IMPUTE2 reports an information metric (info score). This metric typically takes values between 0 and 1, where values near 1 indicate that a SNP has been imputed with high certainty. The info metric is often used to remove poorly imputed SNPs from the association testing results. Different thresholds were recommended for different MAF categories. We considered a SNP with info score great than 0.4 as an acceptable well-imputed variant in this study.

### Concordance analysis

We masked the genotype of one variant at a time throughout the genome in the GWAS data, and then imputed the masked genotypes from the reference data. The imputed genotypes were then compared with the original genotypes to evaluate the quality of the imputation. Only variants with input data were masked and imputed in this analysis, all input genotypes were treated as being true.

For concordance rate analysis, we made hard genotype calls by applying a threshold (0.9) to the maximum value in each input probability triple. For example, an imputed genotype with P(G = 0,1,2) = (0.008, 0.98, 0.012) would be called as a ‘1’ (heterozygous), while a genotype with P(G = 0,1,2) = (0.11, 0.74, 0.15) would be set to missing and omitted from the concordance calculations. The missing rates were low for all the 9 scenarios, the highest missing rate was got at 1KGpilot imputation for 1M array at 1.9% (S1 Table). The missing rates for 317K array were lower than 1M array, because most of the SNPs in 317K array were common SNPs, and were imputable with high probability value (S1 Table).

The squared correlation R2 between input genotypes and expected continuous dosages (not hard call) of each SNP were also reported [17]. To do this, the imputed probability triple should be converted to genotype dosages, for example, for a SNP with P(G = 0,1,2) = (0.008, 0.98, 0.012), the dosage of the reference allele should be 1.004 (= 0.98+0.012*2).

## Results

### Overview of the reference panels

**Figure 1:**
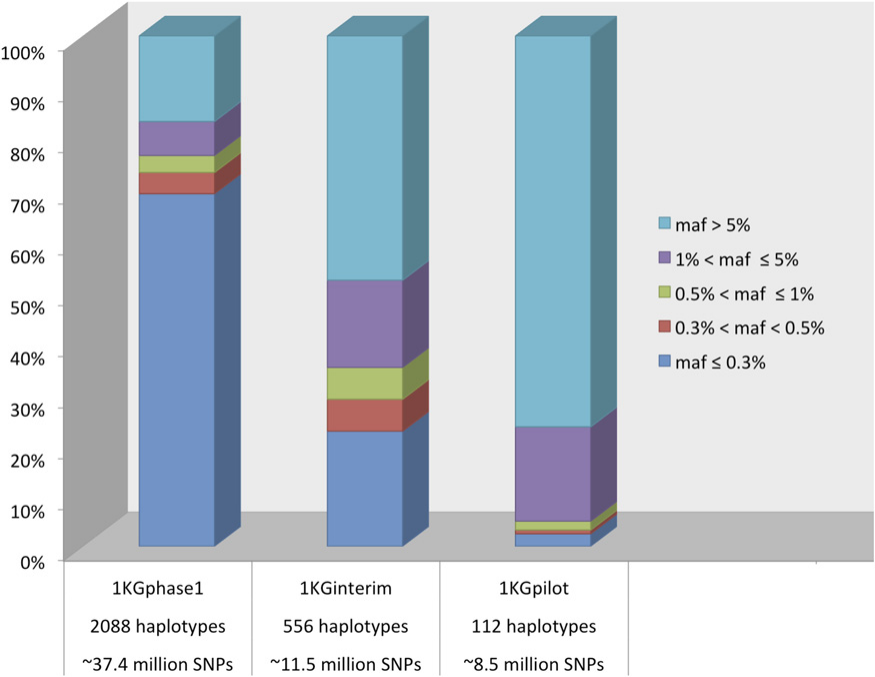
The proportion of variants by Minor Allele Frequency (MAF) across imputation reference panels.

We observed that the sample size and number of variants across the reference panels increases from 1KGpilot to 1KGphase1. Fig. 1 shows the MAF distribution of different reference panels, indicating that most of the variants in 1KGphase1 (including 1094 individuals and ~37.4 million variants) are rare, with a mean MAF of 0.02 (S1 Fig.). Almost half of the variants in the 1KGinterim EUR panel (including 288 EUR individuals and ~11.5 million variants) are common, with mean MAF of 0.12 (S1 Fig.), while the 1KGpilot CEU panel (which includes 56 individuals and ~8.5 million variants) contains mostly common variants and has mean MAF of 0.22 (S1 Fig.).

### Overview of the imputation scenarios

The percentage of well-imputed SNPs (info>=0.4) consistently drops as the mean MAF decreases from 1KGpilot panel to 1KGphase1 panel (Table 1), with the 1KGphase1-based imputations only providing 28% of SNPs with an info score >0.4, however, the absolute number of well-imputed variants increases across the 1000 Genome panels (Table 1). The proportion of the well-imputed SNPs increases while the density of the GWAS genotyped SNPs increased (From 84% to 89% in 1KGpilot-based imputations) (Table 1).

**Table 1:** Overview of the imputation performances for the 3 genome-wide genotype arrays based on different reference panels.

		**1KGpilot CEU (b36) (112 haplotypes, ~8.5M SNPs)**			**1KGinterim EUR (b37) (556 haplotypes, ~11.5M SNPs)**			**1KGphase1 ALL (b37) (2088 haplotypes, ~37.4M SNPs)**		
**GWAS datasets**	Genotyped SNPs*	**Total SNPs**	**Well-imputed SNPs**	**%**	**Total SNPs**	**Well-imputed SNPs**	**%**	**Total SNPs**	**Well-imputed SNPs**	**%**
317k	281,641	8,508,091	7,123,480	84%	11,577,780	7,526,749	65%	37,427,201	10,642,325	28%
610k	488,822	8,510,853	7,412,689	87%	11,581,767	7,767,264	67%	37,427,643	10,649,233	28%
1M	841,995	8,522,561	7,610,312	89%	11,591,081	7,939,987	69%	37,429,304	10,743,754	29%

* SNP QC was done

** Well-imputed SNPs were those with proper info ≥ 0.4

### Imputation concordance of the genotyped variants

The overall concordance rate of different scenarios were reported in Table 2, we can see that the concordance rate is high (up to 93.8%) in all scenarios. Table 3 shows the MAF distribution of the genotyped variants, it is expected that there are few rare variants and some low-frequency variants in the genotyped datasets (Table 3). There are no variants with MAF <0.5%, except only 74 variants in 1M dataset. The squared correlations (R2) between original genotypes and imputed dosages are high even at rare MAF bin (from 0.72 to 0.92 in different scenarios with 0.005<MAF<=0.01) (Table 2), however, the standard deviations of R2 in rare MAF bin are larger than those in common MAF bin (Table 2). The median R2 improves with larger reference panels and higher density of genome-wide genotyping arrays (S2 Fig. and Table 2).

**Table 2:** Concordance of the 9 imputation scenarios.

**GWAS datasets**	**Reference panels**	**Overall concordance rate**	**Median R2 (Std. Dev.)**				
			**MAF<=0.003**	**0.003<MAF<=0.005**	**0.005<MAF<=0.01**	**0.01<MAF<=0.05**	**MAF>0.05**
317K	1KGpilot	93.83	0	0	0.72(0.348)	0.85(0.274)	0.93(0.187)
317K	1KGinterim	93.81	0	0	0.74(0.349)	0.86(0.281)	0.93(0.192)
317K	1KGphase1	94.70	0	0	0.78(0.349)	0.88(0.268)	0.94(0.184)
610K	1KGpilot	96.11	0	0	0.77(0.354)	0.91(0.251)	0.97(0.155)
610K	1KGinterim	96.22	0	0	0.81(0.341)	0.93(0.249)	0.97(0.158)
610K	1KGphase1	96.99	0	0	0.87(0.311)	0.95(0.224)	0.98(0.145)
1M	1KGpilot	97.05	0	0.0015	0.84(0.355)	0.96(0.251)	0.98(0.141)
1M	1KGinterim	97.30	0	0	0.88(0.358)	0.97(0.255)	0.98(0.138)
1M	1KGphase1	97.98	0	0	0.92(0.354)	0.99(0.226)	0.99(0.122)

**Table 3:** The MAF distribution of the genotyped variants.

**GWAS datasets**	Genotyped SNPs*	**MAF<=0.003**	**0.003<MAF<=0.005**	**0.005<MAF<=0.01**	**0.01<MAF<=0.05**	**MAF>0.05**
317k	281,641	0	0	33	7,982	273,626
610k	488,822	0	0	168	20,508	468,146
1m	841,995	0	74	1,203	54,367	786,351

* SNP QC was done

### Imputation Performance by Minor Allele Frequency, Reference Panels and Genotypic Arrays

**Figure 2:**
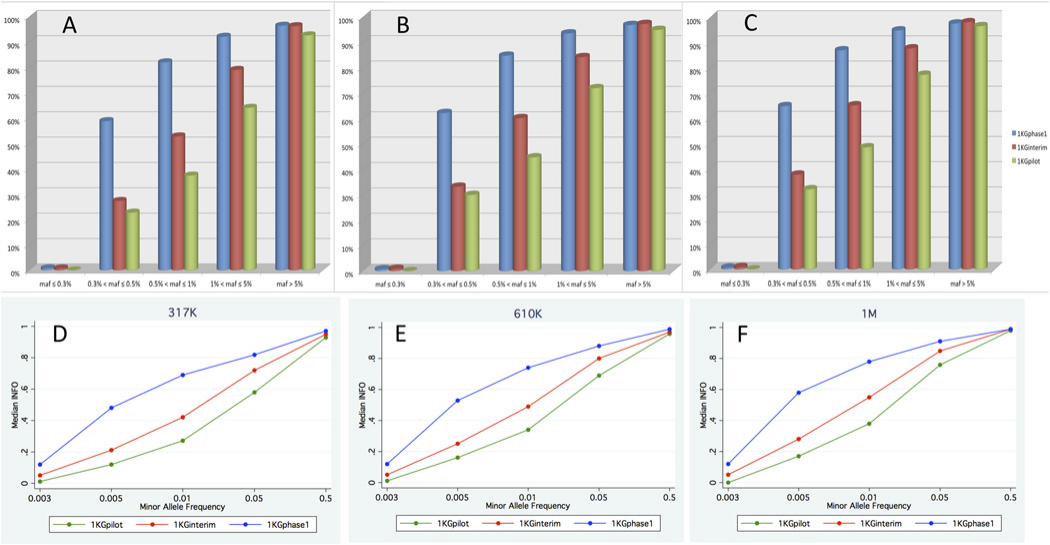
The proportion of well-imputed SNPs (info>0.4) in different MAF bins across imputation reference panels (Panel A is for the 317K genotypic array, Panel B is for 610K genotypic array, and Panel C is for 1M genotypic array). Panel D, E and F is a comparison of median info score across 3 reference panels for 317K, 610K and 1M genotypic array respectively.

Fig. 2B shows the percentage of well-imputed SNPs (info>=0.4) in each MAF bin for 610k-based imputations classified by different reference panels. It shows that common variants are well imputed; more than 95% of the imputed SNPs in common MAF bin had info scores >0.4. For low frequency and rare variants (MAF≤5%), 1KGphase1 outperforms the other reference panels. The proportion of well-imputed SNPs in 610k imputations was 94%, 84%, 72% for SNPs with MAF from 1% to 5% for 1KGphase1, 1KGinterim and 1KGpilot respectively. This proportion of well-imputed SNPs dropped to 85%, 60% and 45% for SNPs with MAF from 0.5% to 1%; and 62%, 33% and 30% for SNPs with MAF from 0.3% to 0.5% for the same reference panels. For SNPs with MAF ≤ 0.3%, only 1% of the variants were well imputed in 1KGphase1 and 1KGinterim imputations, and none are well imputed in 1KGpilot imputation. The 317k/1M imputations show similar performances as 610k, with small decreases in proportion for the 317k array and likewise small increases for the 1M array (Fig. 2A and 2C).

Fig. 2E compares the median info in each MAF bin among the 3 reference panels based upon the 610k array imputations, and consistently demonstrates that 1KGphase1 imputations outperform the other reference panels and this difference is most marked for variants with a MAF <0.05. We note that even with more than ~1000 samples in reference panel such as 1KGphase1, variants with a MAF ≤ 0.003 are not reliably imputed. This frequency range is close the frequency of singletons of the genotype dataset. Similar results are also observed in 317k and 1M imputations (Fig. 2D and 2F).

S3A-C Fig. compare the proportion of well-imputed SNPs in each MAF bin among the 3 GWAS arrays for the 1KGpilot-based, 1KGinterim-based and 1KGphase1-based imputations, respectively. These findings suggest that higher density GWAS genotyping results in small increases in the proportion of well-imputed SNPs. Consistent results were observed when comparing the median info in each MAF bin between the 3 GWAS chip arrays, however, these differences were small compared to effect of the size of the reference panel (S3D-F Fig. vs Fig. 2D–2F). Additionally, the effect of SNP density on imputation quality decreased when the sample size of reference panel increased (S3D-F Fig.).

## Discussion

In this study, we investigated the effect of the size of reference panels and density of genome-wide genotyping arrays on the performance of low frequency and rare variant imputation. Our results demonstrate that the imputation quality of majority of variants with a MAF higher than the frequency of singletons becomes acceptable as the size of the reference panel increased to ~1000 samples such as in the 1KGphase1 release. Very rare variants, such as singletons, are not reliably imputed under any conditions. These results provide guidance in the design and implementation of imputation-based GAW studies.

We note that majority of the common variants (MAF>5%) could be well imputed across all of the 9 scenarios, which is concordant with previously reported results [12, 18, 19, 20]. For low frequency and rare variants (MAF ≤ 5%), 1KGphase1 based imputations consistently outperformed 1KGinterim and 1KGpilot reference panels across all three genome-wide genotyping arrays. This is likely because the sample size of the three 1KG reference panels increased from 56 CEU individuals (1KGpilot) to 283 EUR individuals (1KGinterim) and then to 1094 individuals (1KGphase1). Higher imputation quality as a function of more haplotypes in the reference sample has also been reported for common variant imputation [21]. Another possible reason is that 1KGphase1 panel contains haplotypes from diverse ancestries (EUR, AFR, ASN and AMR), as reported by other researchers, reference panel diversity could increase imputation accuracy to a certain degree either across populations [22, 23] or within the same population [24]. For the very rare variants, the imputation quality is poor across all scenarios, suggesting that imputation of very rare variants will require extremely large reference panel [25] or may be futile [19].

Besides the 1000 Genomes reference panel, more and more large sequencing projects provide public available reference panel for imputation. UK10K consortium (http://www.uk10k.org/) is among one of these, four thousands European-descent samples were whole genome-wide sequenced at 6x depth. Most recently, Marchini et al presented a haplotype map derived from whole genome low-coverage sequencing of over 25,000 individuals at the American Society of Human Genetics meeting in Boston [25], This huge reference panel will be released in the near future. We believe imputation of rare variants will improve as the number of individuals included in reference haplotypes increases.

We also estimated the effect of the density of genotypic arrays on the imputation. The 3 GWAS chip arrays (317K, 610K and 1M) we used in this study were the most common platforms. For the low frequency and rare variants, the imputation quality improved with increasing density of the genotypic array, but the difference is small. These findings provide guidance to cohorts that had previously genome-wide genotyped their samples on older genotypic arrays.

To keep in mind that, INFO score is an estimated quality measurement of imputation. In imputation-based GWAS data analysis, we use INFO score reported by IMPUTE2 as quality control, and keep variants with high score to the downstream analysis. Different thresholds were recommended for different MAF categories [26]. INFO score >0.4 is always used to define a “well-imputed SNP”, however, it is hard to know the imputation accuracy of the imputed SNPs, because there are no “True Genotype” for the SNPs that are not genotyped. Therefore, a better way to avoid the effect of imputation on the association results is to directly genotype the significant variants that came out from the imputation-based analysis. In fact, the statistical power and effect size of association of the variants will improve by doing direct genotyping, as we have commented somewhere else [27].

In our study, we have compared imputation performance only within the IMPUTE2 software [28], similar conclusions that imputation quality increases with larger reference panel sizes could be observed with other imputation programs, at least in MaCH [23, 24].

In summary, the 1000 Genome Project reference panels can be used to impute common, low frequency and rare variants, thereby providing a substantially increased number of variants for analysis. However, the imputation quality for variants with frequency from singletons to 5% is strongly dependent on the sample size of the reference panel, such that the quality increases with the sample size, and with acceptable quality at ~1000 samples such as in the 1KGphase1 release. Genotypic array density also influences the imputation quality. Given the upcoming challenges posed by sequencing studies, our data suggest that imputation quality of rare variants will continue to improve as the number of individuals included in reference haplotypes increases. These data therefore provide guidance in the design and execution of large-scale sequencing based association studies.

## Supporting Information

S1 Fig The MAF distribution of the 3 reference panels. 1KGphase1 has a mean MAF of 0.02 1KGinterim EUR panel has a mean MAF of 0.12; 1KGpilot CEU panel has mean MAF 0.22.
(DOCX)

S2 Fig Panel A, B and C is a comparison of median R2 across 3 GWAS chip arrays for 1KGpilot, 1KGinterim and 1KGphase1 based imputation respectively in different MAF bin. And Panel D, E and F is a comparison of median R2 across the 3 reference panels for 317k, 610k and 1M, respectively. R2 is the squared correlation between input genotypes and imputed dosages.
(DOCX)

S3 Fig The proportion of well-imputed SNPs (info>0.4) in different MAF bins across 3 GWAS chip arrays (Panel A is for 1KGpilot based imputation, Panel B is for 1KGinterim based imputation, and Panel C is for 1KGphase1 based imputation). Panel D, E and F is a comparison of median info score across 3 GWAS chip arrays for 1KGpilot, 1KGinterim and 1KGphase1 based imputation respectively.
(DOCX)

S1 Table For concordance rate analysis, we made hard genotype calls by applying a threshold (0.9) to the maximum value in each input probability triple. A genotype with maximum value less than 0.9 would be set to missing. The missing rates for 317K arrays were lower than 1M array, because most of the SNPs in 317K array were common SNPs, and were imputable with high probability value.
(DOCX)

S1 File The genome-wide genotypes of the 153 samples in PLINK format. (ZIP)
