# Supplementary material for: Performance of Genotype Imputation for Low Frequency and Rare Variants from the 1000 Genomes
Source: PLoS One. 2015 Jan 26;10(1):e0116487. doi: 10.1371/journal.pone.0116487 (PMC4306552; doi:10.1371/journal.pone.0116487)
Supplement: S1 Fig — 1KGphase1 has a mean MAF of 0.02 1KGinterim EUR panel has a mean MAF of 0.12; 1KGpilot CEU panel has mean MAF 0.22. (DOCX) [file pone.0116487.s001.docx]

Figure S1. The MAF distribution of the 3 reference panels. 1KGphase1 has a mean MAF of 0.02; 1KGinterim EUR panel has a mean MAF of 0.12; 1KGpilot CEU panel has mean MAF 0.22.
