# Supplementary material for: Performance of Genotype Imputation for Low Frequency and Rare Variants from the 1000 Genomes
Source: PLoS One. 2015 Jan 26;10(1):e0116487. doi: 10.1371/journal.pone.0116487 (PMC4306552; doi:10.1371/journal.pone.0116487)

Figure S2. Panel A, B and C is a comparison of median R2 across 3 GWAS chip arrays for 1KGpilot, 1KGinterim and 1KGphase1 based imputation respectively in different MAF bin. And Panel D, E and F is a comparison of median R2 across the 3 reference panels for 317k, 610k and 1M, respectively. R2 is the squared correlation between input genotypes and imputed dosages.


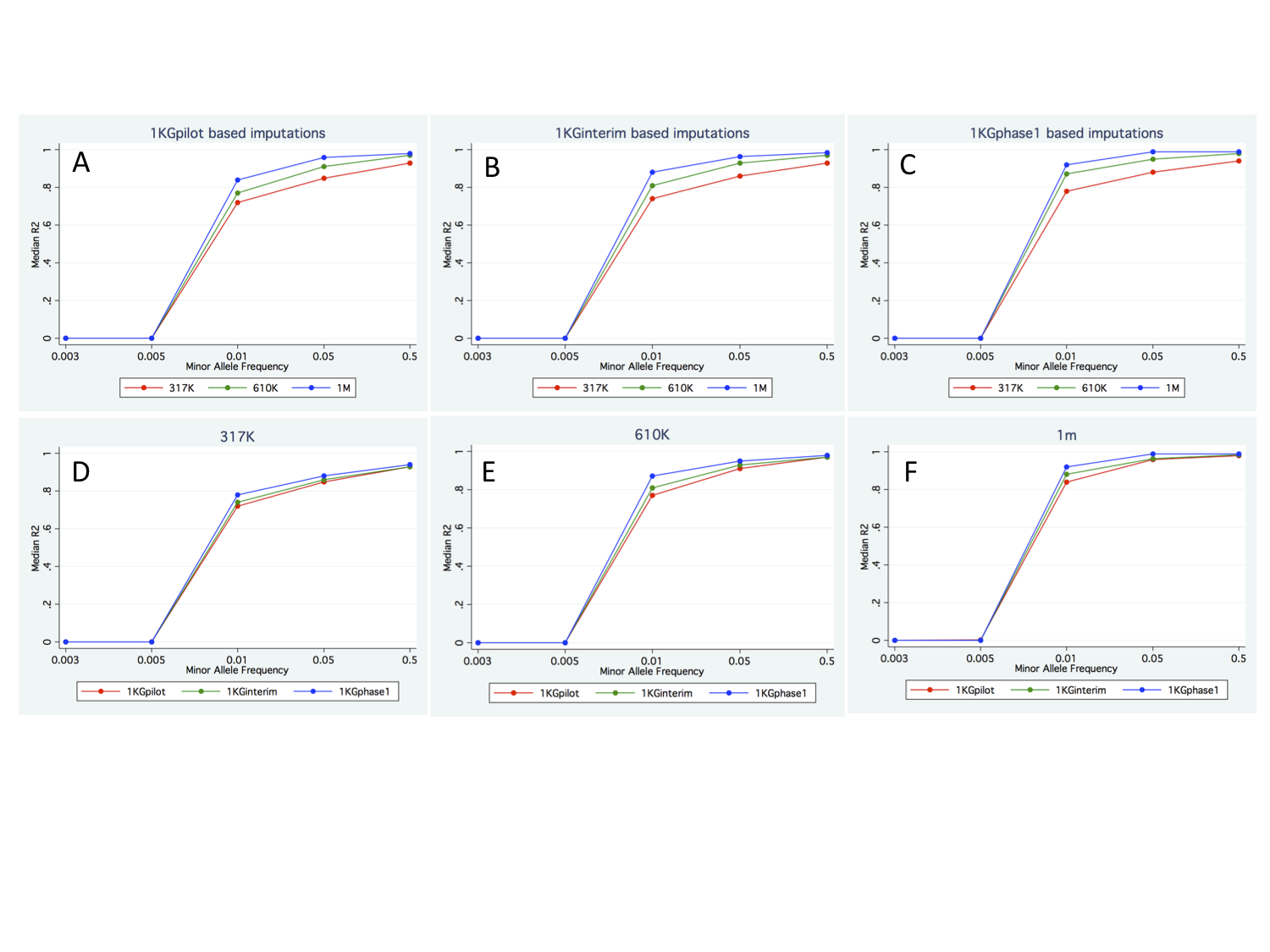

Supplement: S2 Fig — And Panel D, E and F is a comparison of median R2 across the 3 reference panels for 317k, 610k and 1M, respectively. R2 is the squared correlation between input genotypes and imputed dosages. (DOCX) [file pone.0116487.s002.docx]
