# Supplementary material for: Performance of Genotype Imputation for Low Frequency and Rare Variants from the 1000 Genomes
Source: PLoS One. 2015 Jan 26;10(1):e0116487. doi: 10.1371/journal.pone.0116487 (PMC4306552; doi:10.1371/journal.pone.0116487)
Supplement: S3 Fig — Panel D, E and F is a comparison of median info score across 3 GWAS chip arrays for 1KGpilot, 1KGinterim and 1KGphase1 based imputation respectively. (DOCX) [file pone.0116487.s003.docx]

Figure S3. The proportion of well-imputed SNPs (info>0.4) in different MAF bins across 3 GWAS chip arrays (Panel A is for 1KGpilot based imputation, Panel B is for 1KGinterim based imputation, and Panel C is for 1KGphase1 based imputation). Panel D, E and F is a comparison of median info score across 3 GWAS chip arrays for 1KGpilot, 1KGinterim and 1KGphase1 based imputation respectively.
