# Supplementary material for: Performance of Genotype Imputation for Low Frequency and Rare Variants from the 1000 Genomes
Source: PLoS One. 2015 Jan 26;10(1):e0116487. doi: 10.1371/journal.pone.0116487 (PMC4306552; doi:10.1371/journal.pone.0116487)
Supplement: S1 Table — A genotype with maximum value less than 0.9 would be set to missing. The missing rates for 317K arrays were lower than 1M array, because most of the SNPs in 317K array were common SNPs, and were imputable with high probability value. (DOCX) [file pone.0116487.s004.docx]

Table S1. For concordance rate analysis, we made hard genotype calls by applying a threshold (0.9) to the maximum value in each input probability triple. A genotype with maximum value less than 0.9 would be set to missing. The missing rates for 317K arrays were lower than 1M array, because most of the SNPs in 317K array were common SNPs, and were imputable with high probability value.

| GWAS datasets | Reference panels | Missing counts | Missing rate |
| --- | --- | --- | --- |
| 317K | 1KGpilot | 603 | 0.21% |
| 317K | 1KGinterim | 458 | 0.16% |
| 317K | 1KGphase1 | 2 | 0.00% |
| 610K | 1KGpilot | 3544 | 0.73% |
| 610K | 1KGinterim | 2247 | 0.46% |
| 610K | 1KGphase1 | 176 | 0.04% |
| 1M | 1KGpilot | 15999 | 1.90% |
| 1M | 1KGinterim | 12749 | 1.51% |
| 1M | 1KGphase1 | 1090 | 0.13% |
